# Supplementary material for: The interactome of intact mitochondria by cross-linking mass spectrometry provides evidence for coexisting respiratory supercomplexes
Source: Mol Cell Proteomics. 2017 Dec 8;17(2):216–32. doi: 10.1074/mcp.RA117.000470 (PMC5795388; doi:10.1074/mcp.RA117.000470)
Supplement: Supplemental Data [file supp_RA117.000470_133922_0_supp_23978_fzffwf.zip › spectra_annotation/mito_spectra_annotation/35-1-2-1-8-1.pdf]

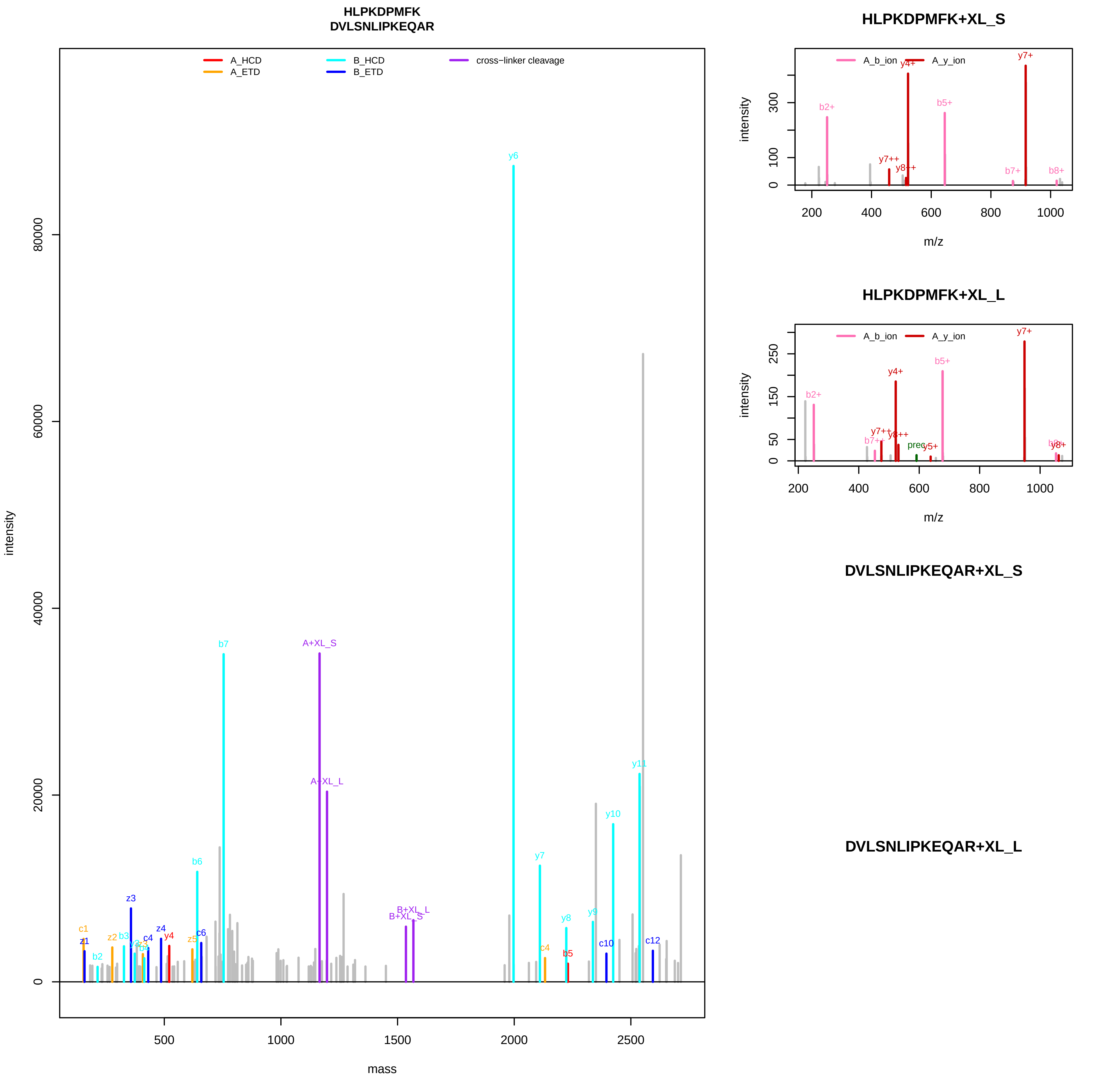
HLPKDPMFK
DVLSNLIPKEQAR
A_HCD
A_ETD
B_HCD
B_ETD
cross−linker cleavage
intensity
mass
0
20000
40000
60000
80000
500
1000
1500
2000
2500
y6
b7
A+XL_S
A+XL_L
b6
B+XL_L
B+XL_S
y7
y8
y9
y10
y11
c10
c12
c4
b5
z3
z4
c1
z1
b2
z2
b3
c4
y4
z5
c6
HLPKDPMFK+XL_S
A_b_ion
A_y_ion
intensity
m/z
0
100
300
200
400
600
800
1000
b2+
y4+
b5+
y7+
y7++
y8++
b7+
b8+
HLPKDPMFK+XL_L
A_b_ion
A_y_ion
intensity
m/z
0
50
150
250
200
400
600
800
1000
b2+
y4+
b5+
y7+
b7++
y7++
y8++
prec
y5+
y8+
DVLSNLIPKEQAR+XL_S
DVLSNLIPKEQAR+XL_L
